# Non-invasive management of severe chlamydia psittaci pneumonia presenting with hypoxemia and diarrhea: a case report

**DOI:** 10.3389/fmed.2026.1855281

**Published:** 2026-08-26

**Authors:** Xuanqi Liu, Dongmei Hu, Jieyi Tan, Yingdian Yu

**Affiliations:** Intensive Care Department, The Affiliated Traditional Chinese Medicine Hospital, Guangzhou Medical University, Guangzhou, China

**Keywords:** atypical clinical manifestation, case report, chlamydia psittaci, high-flow nasal cannula, non-invasive management, omadacycline, severe pneumonia, targeted metagenomic next-generation sequencing

## Abstract

This case report describes a rare presentation of severe Chlamydia psittaci pneumonia in a 43-year-old female patient with prominent hypoxemia and gastrointestinal symptoms, and evaluates the efficacy of standardized non-invasive integrated management for critically ill patients with this atypical phenotype. The patient was admitted with lumbago, persistent high fever, progressive dyspnea, severe hypoxemia, and intractable non-bloody watery diarrhea. Chest computed tomography (CT) revealed extensive bilateral pulmonary ground-glass opacities and consolidation. Rapid and precise etiological diagnosis was achieved via targeted metagenomic next-generation sequencing (mNGS) of bronchoalveolar lavage fluid (BALF), which identified high-load Chlamydia psittaci infection, with 227,155 normalized reads and a genomic coverage of 98.6%. Comprehensive non-invasive multidisciplinary management was implemented throughout the disease course, including high-flow nasal cannula (HFNC) oxygen therapy, dual anti-infective therapy with omadacycline combined with levofloxacin, symptomatic supportive care, and standardized stepwise early rehabilitation training. Dynamic monitoring of clinical and laboratory indicators showed a gradual and sustained decline in inflammatory biomarkers (C-reactive protein,procalcitonin, interleukin-6),accompanied by progressive absorption of pulmonary lesions and recovery of respiratory function. The patient avoided invasive mechanical ventilation throughout hospitalization, was successfully weaned from HFNC on day 14 of admission, and achieved completeclinical, laboratory and radiological recovery at the 1-month follow-up. This case conforms to the CARE (CAse REports) reporting guidelines. It highlights that severe psittacosis pneumonia can present with atypical dominant manifestations of combined hypoxemia and severe gastrointestinal diarrhea, which is easily misdiagnosed clinically. Targeted mNGS enables rapid etiological confirmation of atypical severe psittacosis, and individualized non-invasive integrated management can achieve favorable prognosis in eligible critically ill patients, providing a valuable clinical reference for the standardized diagnosis and treatment of similar rare cases.

## Introduction

Psittacosis, also known as parrot fever, is a zoonotic infectious disease caused by the obligate intracellular pathogen *Chlamydia psittaci*, with avian species as the primary natural reservoir and humans as accidental susceptible hosts (1). Human infection is mainly transmitted via inhalation of aerosolized pathogens from contaminated bird excreta, feathers, and respiratory secretions, posing a persistent public health risk for bird breeders, pet owners, and poultry industry practitioners (2, 3). The disease is distributed globally, with sporadic cases and familial cluster infections increasingly reported in urban areas due to the rising prevalence of pet bird breeding (4).

The clinical manifestations of psittacosis are highly heterogeneous, ranging from asymptomatic latent infection and mild upper respiratory symptoms to severe fatal pneumonia and systemic multi-organ damage (5). Typical clinical features include acute remittent high fever, intractable dry cough, severe intractable headache, generalized myalgia, and extreme fatigue. Critically ill patients may rapidly progress to acute respiratory distress syndrome (ARDS), extrapulmonary organ dysfunction, meningitis, and even death, with significantly elevated mortality in cases of delayed diagnosis and inappropriate treatment (6, 7). A recent Chinese multicenter observational study confirmed that C. psittaci infection accounts for 2.1% of complicated and atypical community-acquired pneumonia cases, with a higher incidence in winter-spring seasons and middle-aged and elderly populations over 50 years old; BALF is verified as the optimal diagnostic specimen for pathogen detection (8). Emerging clinical evidence indicates that prominent gastrointestinal symptoms (persistent diarrhea, nausea, abdominal distension) and early severe hypoxemia are important atypical manifestations of severe psittacosis, and serve as independent predictive factors for poor clinical prognosis (4, 9).

Etiological diagnosis of psittacosis has long been a clinical bottleneck due to its heterogeneous and atypical clinical presentation, as well as the inherent limitations of traditional detection methods. Conventional diagnostic techniques, including pathogen isolation and culture, immunofluorescence assay, complement fixation test, and routine polymerase chain reaction (PCR), are limited by low sensitivity, cumbersome operation, long detection cycle, and dependence on suspected pathogen clues, leading to frequent underdiagnosis, misdiagnosis, and delayed treatment (10). Metagenomic next-generation sequencing (mNGS), especially targeted mNGS (tNGS) for common clinical pathogens, has become a core diagnostic tool for difficult and atypical infectious diseases in recent years. It enables unbiased, rapid, and high-sensitivity identification of rare, fastidious, and difficult-to-culture pathogens such as C. psittaci (11). A authoritative multicenter study confirmed that mNGS achieves a 100% detection sensitivity for C. psittaci infection, which is significantly higher than the 50% sensitivity of conventional qPCR, fully demonstrating its unique advantages in early diagnosis of atypical severe psittacosis (12).

Despite the progress in diagnostic technology, clinical research on standardized management of severe psittacosis pneumonia remains insufficient. Most existing studies focus on patients with typical respiratory-dominant symptoms, with limited reports on comprehensive non-invasive intervention strategies for critically ill patients with combined hypoxemia and severe gastrointestinal manifestations (13, 14). Moreover, the optimal respiratory support mode, individualized anti-infective regimen, and stepwise early rehabilitation protocol for severe psittacosis without invasive ventilation have not been standardized, resulting in inconsistent clinical management practices. The main risk factors for severe progression of psittacosis include advanced age, immune dysfunction, delayed etiological diagnosis, and extrapulmonary organ involvement (4, 15).

The study population applicable to this non-invasive integrated management strategy is defined as adult patients with severe psittacosis pneumonia complicated by acute hypoxemic respiratory failure, who do not meet clinical endotracheal intubation indications, without irreversible respiratory collapse, refractory hypoxemia, or multiple organ dysfunction syndrome. Such patients are suitable for HFNC respiratory support and standardized comprehensive non-invasive intervention, which is the core applicable scope of the clinical strategy proposed in this study.

Against this background, we report a typical atypical case of severe psittacosis pneumonia in a 43-year-old female patient with simultaneous prominent hypoxemia and intractable watery diarrhea. We systematically describe the clinical characteristics, tNGS-based etiological diagnostic process, and full-course non-invasive integrated management scheme of the case. By dynamically analyzing clinical symptoms, laboratory biomarkers, and imaging changes throughout hospitalization, we validate the high clinical value of targeted mNGS in the diagnosis of atypical severe psittacosis, and explore the feasibility and efficacy of standardized non-invasive intervention to avoid invasive mechanical ventilation in critically ill patients. This study aims to supplement the clinical phenotypic spectrum of psittacosis and provide practical, standardized clinical references for the individualized diagnosis and treatment of similar atypical severe cases.

### Case description

Day 1 (admission): 5-day persistent high fever, lumbago, 2-day progressive dyspnea, accompanied by severe hypoxemia and non-bloody watery diarrhea; Day 2: bronchoscopy was performed to collect BALF for targeted mNGS detection; Day 3: fever subsided, diarrhea completely resolved, standardized stepwise early rehabilitation initiated; Day 7: inflammatory markers decreased significantly, oxygenation index improved continuously; Day 10: chest CT showed obvious absorption of bilateral pulmonary inflammatory lesions; Day 14: successful weaning from HFNC oxygen therapy; Day 16: stable vital signs, transferred to general ward; 2-week and 1-month follow-up: complete recovery of clinical symptoms, laboratory indicators, and pulmonary imaging, without residual complications.

A 43-year-old female patient was admitted to the Department of Respiratory and Critical Care Medicine with the chief complaint of “lumbago and persistent high fever for 5 days, progressive dyspnea for 2 days”. The patient had a 3-year history of pet parrot breeding, with no recent bird purchase, death, or abnormal morbidity, and denied contact with other poultry, pigeons, bird markets, and farms. She also denied consumption of raw or undercooked poultry and other high-risk dietary exposures, excluding common food-borne causes of diarrhea. Five days before admission, she developed sudden lumbago (visual analog scale score: 6/10) and sustained high fever (maximum body temperature: 39.8 °C), accompanied by non-bloody watery diarrhea 4, 5 times per day, without abdominal pain, tenesmus, or mucus in stool. Two days after symptom onset, progressive activity-induced dyspnea occurred and gradually aggravated, with ineffective symptomatic treatment in a local clinic, so she was transferred to our hospital for further treatment.

Neurological and autonomic nervous system assessment: throughout the disease course, the patient had clear consciousness, no headache, dizziness, nausea, vomiting, blurred vision, limb numbness and weakness, convulsions, or disturbance of consciousness. There were no abnormal autonomic nervous system manifestations such as persistent sweating, abnormal heart rate fluctuation, blood pressure instability, gastrointestinal peristalsis disorder (except disease-related diarrhea), or urinary dysfunction, indicating no neurological or autonomic nervous system involvement in this case.

On admission, vital signs were as follows: body temperature 39.2 °C, heart rate 118 beats/min, respiratory rate 32 breaths/min, blood pressure 126/78 mmHg, room air oxygen saturation (SpO_2_) 88%. Physical examination showed slightly cyanotic lips, bilateral lower lung fine rales, and normal abdominal physical examination, without neurological positive signs.

Routine admission laboratory tests, inflammatory biomarkers, and biochemical indexes were detected, with detailed results and clinical normal reference ranges shown in Table 1. Sputum, blood, and stool routine cultures were all negative, which was consistent with the biological characteristics of C. psittaci (obligate intracellular parasite, unable to grow in conventional artificial culture media).

**Table 1:** Dynamic changes of main laboratory indicators and corresponding normal reference ranges during hospitalization.

Laboratory parameter	Normal reference range	Day1 April 1	Day3 April 3	Day7 April 5	Day9 April 7	Day11 April 9	Day13 April 11	Day15 April 13
Leucocytes (10^9^/L)	3.5–9.5	6.40	5.82	6.01	5.10	6.04	8.67	–
hsCRP (mg/L)	0.0–0.1	>200	196.4	>200	>200	37.3	12.9	–
Neutrophils (%)	40.0–75.0	88.9	93.4	93.9	79.3	77.7	79.1	–
Lymp*hoc*yte (%)	20.0–50.0	8.4	4.8	3.8	12.5	14.9	15.2	–
Erythrocytes (10^12^/L)	3.80–5.10	2.97	2.39	2.46	2.23	2.49	2.23	–
Hemoglobin (g/L)	115–150	95	76	77	71	78	70	–
Platelets (10^9^/L)	125–350	95	77	128	253	347	399	–
ALT (U/L)	7.0–40.0	51.5	–	249.1	189.2	137.8	–	–
AST (U/L)	13.0–35.0	89.1	93.6	328.8	157.1	73.8	–	–
CK (U/L)	40.0–200.0	*2,037*.2	*2,250*.9	–	167.6	100.2	–	–
CK-MB (ng/mL)	* ≤* 5.00	2.12	2.81	–	1.18	1.28	–	–
BUN (mmol/L)	2.60–7.50	3.46	–	–	–	–	–	–
Creatinine (umol/L)	41.0–73.0	62.3	–	–	–	–	–	–
ALB (g/L)	35.0–50.0	37.4	–	–	33.1	33.4	–	–
Total bilirubin (umol/L)	5.0–21.0	18.8	–	–	–	15.1	–	–
Direct bilirubin (umol/L)	* ≤* 5.10	7.8	–	–	–	6.7	–	–
MYO (ng/mL)	< 110.0	622.96	107.29	–	–	–	–	–
BNP (pg/mL)	< 125.0	265.0	*1,430*	*1,891*.0	337.0	86.0	–	–
Oxygenation Index	400–500	120	143	216	236	268	364	–
PaC*O_2_* (mmHg)	35.0–45.0	35.3	31.3	31.2	33.1	32.7	32.1	–
P*T(*s)	9.4–12.5	13.6	12.3	13.6	–	–	–	11.0
APP*T(*s)	25.1–36.5	41.8	38.3	29.8	–	–	–	34.9
Dimer (mg/I FEU)	* ≤* 0.50	–	2.15	5.79	7.25	–	5.42	2.0
Temperature (*°C*)	36.0–37.2	40.1	38.8	38.7	37.3	37.0	36.9	36.7

hsCRP, High-Sensitivity C-reactive protein; ALT, Alanine Aminotransferase; AST, Aspartate Aminotransferase; CK, creatine kinase. The symbol “-” indicates no laboratory detection was performed on the corresponding day of hospitalization.

**Figure 1:**
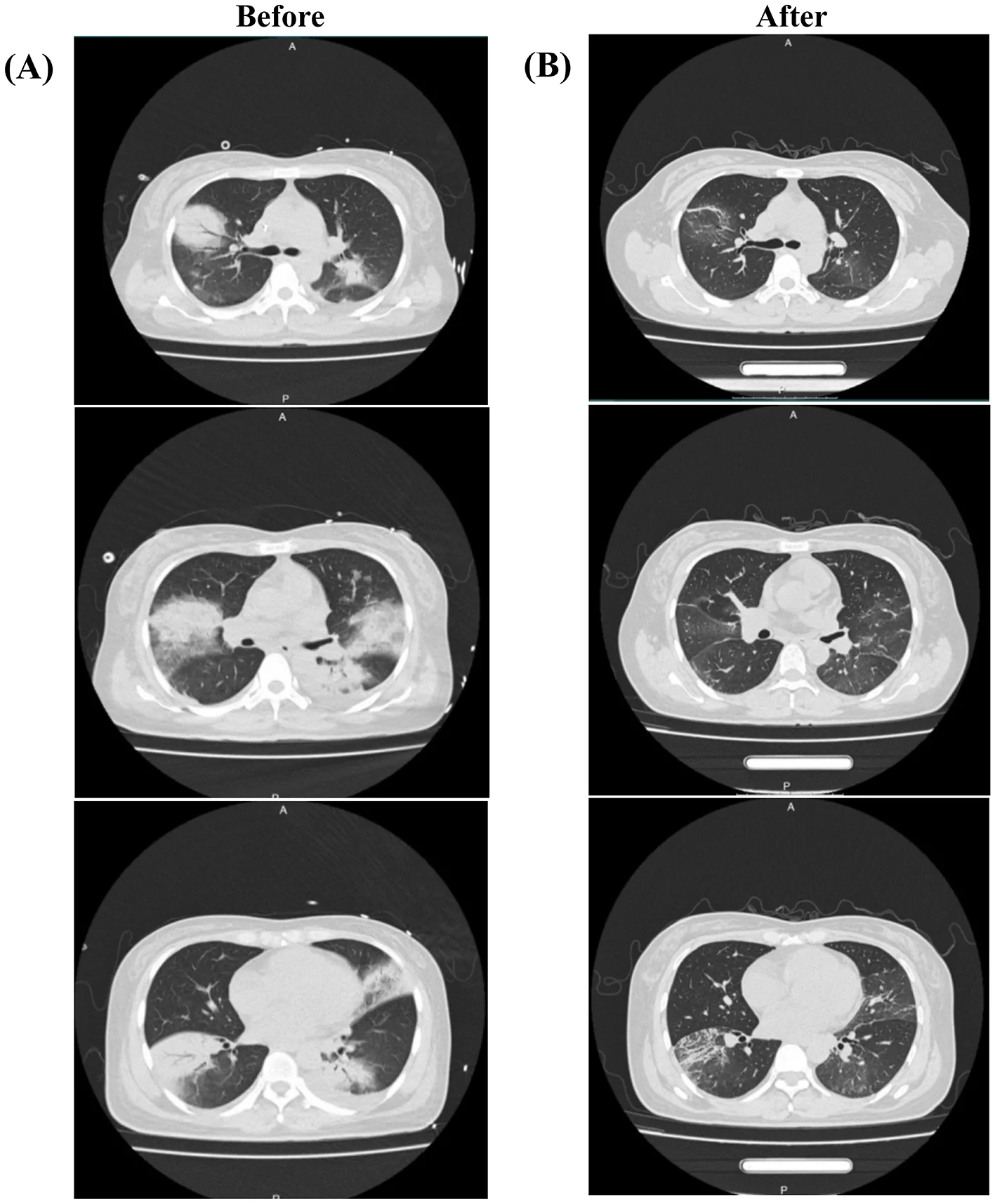
Radiological imaging examinations during hospitalization.

Chest plain CT scan on admission showed extensive patchy ground-glass opacities and consolidation shadows in bilateral upper and lower lung lobes, with uneven lung density, consistent with the imaging manifestations of severe community-acquired pneumonia (Figure 1).

To clarify the pathogenic etiology, flexible bronchoscopy was performed on the 2nd day of admission to collect BALF specimens for 265 kinds of common pathogens targeted next-generation sequencing (tNGS, BGI Genomics, Shenzhen, China). The tNGS detection was strictly implemented in accordance with standardized experimental procedures, with qualified internal quality control, external quality control, and Q30 base quality evaluation (Q30 ratio met clinical detection standards, sequencing error rate < 0.1%).

Detailed mNGS technical parameters: total valid sequencing reads of the specimen were up to standard, with a Q30 proportion of qualified level. The detection limit of the platform was 50 copies/mL. The results showed that Chlamydia psittaci was the dominant pathogenic bacterium, with 227,155 normalized reads, an estimated pathogen concentration of 106 copies/mL, and a genomic coverage of 98.6%. Multiple conditional pathogenic bacteria (Morganella morganii, Klebsiella pneumoniae, Pseudomonas aeruginosa, etc.,) were detected at low abundance, which were judged as colonizing background bacteria combined with clinical manifestations, culture results, and imaging features, without clinical pathogenic significance. No fungi, viruses, or parasites were detected. Subsequent serological test confirmed positive C. psittaci IgM antibody (Titer 1:320), which comprehensively confirmed the etiological diagnosis of severe C. psittaci pneumonia.

The final clinical diagnosis was severe psittacosis pneumonia complicated by acute hypoxemic respiratory failure and gastrointestinal dysfunction. A full-course standardized non-invasive integrated management strategy was immediately initiated:

1. Non-invasive respiratory support: high-flow nasal cannula oxygen therapy was applied with an initial flow rate of 40 L/min and oxygen concentration of 50%, dynamically adjusted according to SpO_2_ and patient tolerance, maintaining peripheral oxygen saturation at 93–96%.

2. Targeted dual anti-infective therapy: omadacycline (100 mg, intravenous infusion, q12h) combined with levofloxacin (500 mg, intravenous infusion, qd), with a standardized treatment course of 14 days. The regimen was selected based on the high antibacterial activity of two drugs against C. psittaci and broad-spectrum coverage of common community-acquired pneumonia pathogens.

3. Symptomatic supportive treatment: regular antipyretic therapy (paracetamol 500 mg, oral administration, prn) for persistent high fever; antidiarrheal treatment (loperamide 2 mg, oral administration, q12h) to improve gastrointestinal symptoms; and individualized enteral and parenteral nutritional support to maintain internal environment stability.

4. Stepwise early rehabilitation training: starting from the 3rd day of admission after fever control and diarrhea relief, standardized bed-based rehabilitation was carried out, including deep breathing training, effective cough training, and passive and active limb exercise. After oxygenation index stabilized, gradual off-bed ambulation training was added to improve lung ventilation function and prevent ICU-acquired weakness.

Dynamic monitoring of clinical laboratory indicators and imaging changes was performed throughout the treatment course, and the patient's condition improved progressively. On the 3rd day of treatment, the patient's body temperature returned to normal, diarrhea symptoms completely resolved, and SpO_2_ stabilized at 95−98% with HFNC flow rate adjusted to 35 L/min. On the 7th day, inflammatory biomarkers decreased significantly (CRP 38.2 mg/L, PCT 0.32 ng/mL, IL-6 46 pg/mL). Chest CT re-examination on the 10th day showed significant absorption of bilateral pulmonary inflammatory lesions. On the 14th day, the patient was successfully weaned from HFNC and switched to conventional nasal cannula oxygen therapy (2 L/min), with SpO_2_ maintained at 97−99% during rest and mild activity.

The patient was transferred to the general ward on the 16th day of admission, without complications such as ARDS, sepsis, and multiple organ failure. During follow-up, the patient had mild anxiety in the early stage after discharge due to critical illness and zoonotic infection diagnosis, which was completely relieved after psychological counseling. At the 1-month follow-up, chest CT showed complete absorption of pulmonary lesions, respiratory function returned to normal, and the patient fully resumed daily life and work without post-traumatic stress disorder or residual organ dysfunction.

## Discussion

Psittacosis is a neglected zoonotic infectious disease with diverse and variable clinical phenotypes. Delayed diagnosis and intervention can easily lead to severe pneumonia and systemic inflammatory response, especially in middle-aged patients with bird exposure history (9, 16). This case report innovatively describes a severe psittacosis case with severe persistent watery diarrhea and early refractory hypoxemia as the core initial manifestations, which is significantly different from the traditional fever-headache dominant phenotype. We systematically verify the high diagnostic value of standardized targeted mNGS in atypical severe psittacosis, and confirm the clinical efficacy and safety of full-course non-invasive integrated management for critically ill patients, providing new insights for the clinical diagnosis and treatment of psittacosis.

The most prominent highlight of this case is its unique atypical clinical phenotype. Typical psittacosis is dominated by systemic toxic symptoms such as high fever and severe headache, with mild or occasional gastrointestinal discomfort as secondary accompanying symptoms (3, 4). Epidemiological data show that the overall incidence of gastrointestinal symptoms in psittacosis patients is only 15.6%, mostly manifested as mild nausea and intermittent abdominal distension, and persistent severe watery diarrhea is extremely rare (9). In this case, the 43-year-old female patient presented with intractable watery diarrhea (4, 5 times/day) at the early stage of the disease, accompanied by progressive severe hypoxemia, without typical severe headache symptoms, which greatly increased the difficulty of early clinical differential diagnosis. The causality between diarrhea and C. psittaci infection was fully confirmed: the patient had no dietary or environmental exposure history of enteric pathogens, all stool and blood cultures were negative, mNGS excluded other intestinal pathogens, and diarrhea symptoms were completely relieved synchronously with targeted anti-psittacosis treatment, confirming that gastrointestinal dysfunction was an extrapulmonary manifestation of C. psittaci infection rather than complicated intestinal infection. In addition, neurological and autonomic nervous system involvement was completely excluded in this case, which supplements the clinical blank of phenotypic characteristics of severe psittacosis without neural system damage. Clinically, for patients with clear bird exposure history, unexplained severe hypoxemic pneumonia combined with intractable diarrhea, C. psittaci infection should be included in the priority differential diagnosis list to avoid missed diagnosis and misdiagnosis.

To further clarify the clinical characteristics of atypical psittacosis with gastrointestinal manifestations, we systematically searched and summarized recent domestic and foreign related case reports and case series, and compared the clinical characteristics of typical reported cases with gastrointestinal symptoms and the present case (Table 2). Compared with previous studies, the gastrointestinal symptoms of our case are more severe and persistent, which are the core initial symptoms rather than mild secondary manifestations. Meanwhile, the combination of early severe hypoxemia and persistent diarrhea forms a unique atypical phenotype, which is different from the existing cases with mild gastrointestinal symptoms or late respiratory deterioration. More importantly, this case achieved complete recovery through standardized non-invasive multidisciplinary management, successfully avoiding invasive mechanical ventilation, which provides a safer and more economical treatment scheme for severe atypical psittacosis patients who do not meet intubation criteria, and makes up for the lack of non-invasive management experience in previous similar cases.

**Table 2:** Comparison of clinical characteristics of psittacosis pneumonia with gastrointestinal manifestations in recent studies.

References	Age/ gender	Main gastrointestinal symptoms	Respiratory manifestations	Severity	Diagnostic method	Outcome
Veletzky et al. (9)	52/F	Mild nausea, occasional diarrhea	Moderate cough, no obvious hypoxemia	Moderate pneumonia	mNGS	Full recovery
Ni et al. (3)	58/M	Abdominal distension, intermittent diarrhea	Progressive dyspnea, mild hypoxemia	Severe pneumonia	mNGS + serology	Full recovery
Duan et al. (18)	47/M	Mild vomiting, no diarrhea	Severe dyspnea, refractory hypoxemia	Severe pneumonia	mNGS	Improved with invasive ventilation
Present case	43/F	Persistent severe watery diarrhea (4-5 times/day), no abdominal pain	Early-onset progressive dyspnea and severe hypoxemia	Severe pneumonia	mNGS + IgM antibody	Full recovery with non-invasive management

The standardized non-invasive integrated management strategy adopted in this case is scientific and effective, forming a complete set of clinical intervention models for severe atypical psittacosis. For severe hypoxemic respiratory failure caused by psittacosis, early HFNC oxygen therapy can effectively improve pulmonary oxygenation, reduce respiratory muscle workload, avoid oxygenation deterioration and endotracheal intubation, and is the preferred non-invasive respiratory support mode for such patients (7). The dual anti-infective regimen of omadacycline combined with levofloxacin has significant synergistic antibacterial effect: omadacycline has high tissue permeability and can accumulate at high concentration in pulmonary epithelial lining fluid, targeting intracellular C. psittaci; levofloxacin has broad-spectrum antibacterial activity, covering potential combined bacterial infections, which is consistent with the optimal anti-infective scheme for severe psittacosis recommended in current guidelines (6, 10). In addition, the stepwise early rehabilitation training implemented in this case effectively promotes the absorption of pulmonary inflammatory lesions, improves lung ventilation function, prevents ICU-acquired weakness and muscle atrophy, and shortens the overall hospitalization cycle, which is a valuable supplement to the comprehensive treatment system of severe psittacosis.

From the perspective of immune pathogenesis, dynamic changes of inflammatory biomarkers in this case fully reflect the effectiveness of treatment. The patient's initial IL-6 level was as high as 386 pg/mL, indicating severe systemic inflammatory storm induced by C. psittaci infection. After targeted anti-infective and symptomatic treatment, IL-6 decreased rapidly to 46 pg/mL on the 7th day, and CRP and PCT gradually returned to normal levels, indicating that the inflammatory cascade reaction was effectively inhibited and immune homeostasis was restored (9). The dynamic improvement of oxygenation index and imaging lesions was highly consistent with the decline of inflammatory indicators, verifying that monitoring inflammatory biomarkers can accurately evaluate disease progression and therapeutic efficacy.

Targeted mNGS is the cornerstone of accurate early diagnosis in this case, making up for all defects of traditional detection methods. Traditional C. psittaci culture requires strict biosafety conditions and special culture medium, with a long detection cycle and extremely low positive rate; routine qPCR detection relies on targeted pathogen suspicion and presents limited diagnostic sensitivity, while serological antibody detection can only be used for retrospective diagnosis, failing to guide early clinical intervention (17). A recent authoritative multicenter retrospective study focusing on psittacosis diagnosis systematically compared the diagnostic efficacy of mNGS and conventional qPCR, verifying that standard clinical mNGS achieves a 100% detection sensitivity for C. psittaci infection, which is significantly higher than the 50% sensitivity of conventional qPCR (*p* < 0.01) (12). Consistent with this literature conclusion, the targeted mNGS adopted in our case using standard clinical sequencing datasets exhibited ultra-high sensitivity and specificity, with a pathogen genomic coverage of 98.6% and a high pathogen load of 106 copies/mL, enabling rapid and accurate confirmation of C. psittaci etiology within 48 h after admission. Notably, previous studies have confirmed that BALF is the optimal specimen for C. psittaci mNGS detection, with significantly higher detection positive rate and pathogen reads abundance than blood specimens, which further supports the rationality of BALF-based tNGS sampling in our case (12). Additionally, relevant research has proven that expanded sequencing datasets cannot improve the diagnostic efficiency of C. psittaci mNGS, instead leading to increased human DNA proportion and reduced microbial effective reads, further validating the reliability of our standard clinical tNGS detection scheme. Rapid and precise etiological diagnosis via tNGS enabled timely initiation of targeted anti-infective therapy, avoided the abuse of broad-spectrum antibiotics, and greatly reduced the risk of disease progression and adverse complications in this severe atypical case (15, 19–21).

This study has certain limitations. First, it is a single-case report with limited population representativeness, and the conclusion needs to be verified by multicenter and large-sample prospective studies. Second, only 1-month short-term follow-up data was collected, and long-term follow-up is still needed to observe whether there are residual pulmonary fibrosis and other long-term complications. Third, the specific molecular mechanism of severe gastrointestinal symptoms in psittacosis infection has not been fully clarified, which needs further basic experimental research. Future studies will focus on designing controlled clinical trials to compare the efficacy of different respiratory support modes and anti-infective regimens, and explore early warning biomarkers for severe progression of psittacosis, so as to form a standardized and unified diagnosis and treatment system for atypical psittacosis pneumonia.

In conclusion, severe Chlamydia psittaci pneumonia can present with atypical dominant manifestations of combined severe hypoxemia and intractable gastrointestinal diarrhea, without neurological and autonomic nervous system involvement. Targeted mNGS is an efficient and accurate tool for early etiological diagnosis of such atypical severe cases. The non-invasive integrated management model composed of HFNC oxygen therapy, omadacycline combined with levofloxacin targeted anti-infection, symptomatic supportive care, and stepwise early rehabilitation can achieve excellent clinical prognosis in eligible critically ill patients, effectively avoiding invasive mechanical ventilation. This case supplements the clinical phenotypic spectrum of psittacosis, and provides a standardized, operable clinical reference for the accurate diagnosis and individualized non-invasive treatment of severe atypical psittacosis pneumonia.

## Data Availability

The datasets presented in this study can be found in online repositories. The names of the repository/repositories and accession number(s) can be found in the article/supplementary material.
